# The association between nonsuicidal self-injury and cyberbullying

**DOI:** 10.1192/j.eurpsy.2026.10576

**Published:** 2026-07-31

**Authors:** B. F. Farkas, J. Balázs, D. Győri, D. Komáromy, N. Kollárovics, P. Garas, L. O. Horváth

**Affiliations:** 1 Department of Psychiatry and Psychotherapy, Semmelweis University; 2 Institute of Psychology, Eötvös Loránd University, Budapest, Hungary; 3 Oslo New University College, Oslo, Norway; 4 Doctoral School of Psychology, Eötvös Loránd University, Budapest, Hungary; 5 Department of Political Science, Universiteit van Amsterdam, Amsterdam, Netherlands; 6 Doctoral School, Semmelweis University; 7 Pedagogical Services, Budapest, Hungary

## Abstract

**Introduction:**

Although both nonsuicidal self-injury (NSSI) and cyberbullying (CB) are very common among adolescents, there are only a few studies examining the relationship between the two phenomena.

**Objectives:**

The aim of our study was to explore the relationship between CB and NSSI, and to examine the potential mediating effects of anxiety and depression.

**Methods:**

119 Hungarian adolescents (70% female, 13–18-year-olds) were involved in the study. A self-report questionnaire about CB, the Inventory of Statements About Self-injury, and the Mini International Neuropsychiatric Interview – Kid were administered. Regression and network analysis were conducted to analyze the interrelationships among CB, NSSI, and anxiety/depression.

**Results:**

The prevalence of NSSI was significantly higher among those who were involved in CB, compared to those who were not, χ2(1) = 5.62, p = 0.02. CB and NSSI were mediated by anxiety, and anxiety disorder was directly related to NSSI. Conversely, depression was not directly related to CB, only to anxiety. Victimization was positively related to anxiety, which, in turn, was positively associated with NSSI.

**Conclusions:**

Our results draw attention to the importance of focusing on comorbid anxiety disorders and to both those who are involved in CB and those who witness CB in NSSI prevention strategies.

**Disclosure of Interest:**

None Declared

